# Identification of Pectin Degrading Enzymes Secreted by *Xanthomonas oryzae* pv. *oryzae* and Determination of Their Role in Virulence on Rice

**DOI:** 10.1371/journal.pone.0166396

**Published:** 2016-12-01

**Authors:** Lavanya Tayi, Roshan V. Maku, Hitendra Kumar Patel, Ramesh V. Sonti

**Affiliations:** CSIR-Centre for Cellular and Molecular Biology, Hyderabad, Telangana State, India; University of Nebraska-Lincoln, UNITED STATES

## Abstract

*Xanthomonas oryzae* pv.*oryzae* (Xoo) causes the serious bacterial blight disease of rice. Xoo secretes a repertoire of plant cell wall degrading enzymes (CWDEs) like cellulases, xylanases, esterases etc., which act on various components of the rice cell wall. The major cellulases and xylanases secreted by Xoo have been identified and their role in virulence has been determined. In this study, we have identified some of the pectin degrading enzymes of Xoo and assessed their role in virulence. Bioinformatics analysis indicated the presence of four pectin homogalacturonan (HG) degrading genes in the genome of Xoo. The four HG degrading genes include one polygalacturonase (*pglA*), one pectin methyl esterase (*pmt*) and two pectate lyases (*pel* and *pelL*). There was no difference in the expression of *pglA*, *pmt* and *pel* genes by laboratory wild type Xoo strain (BXO43) grown in either nutrient rich PS medium or in plant mimic XOM2 medium whereas the expression of *pelL* gene was induced in XOM2 medium as indicated by qRT-PCR experiments. Gene disruption mutations were generated in each of these four genes. The polygalacturonase mutant *pglA*^*-*^ was completely deficient in degrading the substrate Na-polygalacturonicacid (PGA). Strains carrying mutations in the *pmt*, *pel* and *pelL* genes were as efficient as wild type Xoo (BXO43) in cleaving PGA. These observations clearly indicate that PglA is the major pectin degrading enzyme produced by Xoo. The pectin methyl esterase, Pmt, is the pectin de-esterifying enzyme secreted by Xoo as evident from the enzymatic activity assay performed using pectin as the substrate. Mutations in the *pglA*, *pmt*, *pel* and *pelL* genes have minimal effects on virulence. This suggests that, as compared to cellulases and xylanases, the HG degrading enzymes may not have a major role in the pathogenicity of Xoo.

## Introduction

The plant cell wall is a structural barrier that is mostly composed of polysaccharides such as cellulose, various hemicelluloses and pectin. The pectin component includes homogalacturonan (HG), xylogalacturonan (XGA), apiogalacturonan, rhamnogalacturonan I (RGI) and rhamnogalacturonan II (RGII). The ratio between these various pectic polysaccharides is variable but often HG is the most abundant component [1]. Based on their activities, the enzymes which are involved in degrading HG are grouped into three classes. The pectin methylesterases de-esterify pectin. The de-esterified pectin is now available for degradation by enzymes that degrade polygalacturonic acid (PGA). These enzymes are polygalacturonases and pectate lyases. Polygalacturonases catalyse a hydrolytic cleavage while lyases act through transelimination mechanism. Pectin lyases and pectate lyases cleave natural pectin (PGA that is methyl esterified) and PGA respectively [2].

Pectin degrading enzymes are secreted by many bacterial and fungal pathogens of plants. In several such pathogens they are demonstrated to be important for full virulence[3–5]. Biochemical and functional aspects of pectinases have been studied extensively in pathogens which cause host tissue maceration like those belonging to genera *Erwinia* and *Ralstonia*. Pectolytic enzymes are the principal virulence factors in these soft rot disease causing pathogens. In these pathogens, mutations in genes encoding pectolytic functions results in reduced virulence. Three polygalacturonases (namely PehA, PehB & PehC) and a pectin methyl esterase (Pme) are encoded in the genome of *Ralstonia solanacearum*. The PehA protein is an endopolygalacturonase while PehB and PehC are exopolygalacturonases. Mutations in the *pehC* and *pme* genes do not affect virulence of *R*. *solanacearum*. Mutations in *pehA* and *pehB* cause reduced virulence. These studies show, that polygalacturonase activity contributes to virulence in this bacterium [6–9]. Interestingly, the *pehA*^*-*^ *pehB*^*-*^ *pehC*^*-*^ triple mutant is much more virulent than the wild type strain. It has been suggested that this increase in virulence can be due to the absence of degradation products of pectin that can serve as inducers of plant defense responses [6, 10]. A quadruple mutant of *Erwinia chrysanthemi* that is deficient in four different pectate lyases displayed reduced maceration (an indication of reduced virulence) on potato tubers [11].

Although Xanthomonads do not cause much tissue maceration, pectolytic ability is shown by some strains like *Xanthomonas campestris* pv. *vescicatoria* (Xcv), *Xanthomonas axonopodis* pv. *citri* (Xac), *Xanthomonas campestris* pv. *campestris* (Xcc) etc. In Xcc, the presence of pectate lyases and their role in virulence has been reported. A mutation in a polygalacturonate lyase was shown to be not required for black rot pathogenesis in turnip (*Brassica campestris*) [12]. Two polygalacturonase genes (*pehA* and *pglA)* and five pectate lyase genes *(pelA1*, *pelA2*, *pelB*, *hrpW* and *pelE*) have been annotated in the genomes of Xcc strains ATCC 33913 and 8004 [13, 14]. Three pectate lyase genes (*pel*, degenerated *pel* and *pelB*) and two polygalacturonase genes (*peh1* and *pglA*) are encoded in the genome sequence of Xac 306 [14]. The role of some of these pectic enzymes in pathogenesis of Xcc and Xac is also determined. In Xac, pectate lyase activity is associated with water soaking symptoms [15]. In Xcc, the *pehA* gene encodes the major polygalacturonase. As tested in cabbage seedlings, mutational analysis suggested that PehA plays a minor role in Xcc virulence [2]. PelA1 is the major pectate lyase of Xcc and like PehA, its expression is regulated by global regulators such as Clp and RpfF [16].

*Xanthomonas oryzae* pv. *oryzae* (Xoo), the causal agent of bacterial leaf blight disease secretes various cell wall degrading enzymes using the type two secretion system. Some of the key cell wall degrading enzymes like a cellulase (ClsA), cellobiosidase (CbsA), xylanase (Xyn) and an esterase (LipA) were identified and found to be required for full virulence of Xoo on rice [17,18]. Till date, the pectin degrading enzymes secreted by Xoo have not been identified. In this study, we have identified a pectin methyl esterase (Pmt) and a polygalacturonase (PglA) as the pectin de-esterifying and pectin degrading enzymes secreted by Xoo. We also show that pectinase deficient mutants are minimally affected for virulence on rice.

## Materials and Methods

### Bacterial strains, plasmids, primers and culture media used

Bacterial strains and plasmids used in the study are listed in Table 1. Primers used in the study are listed in S1 Table. *E*. *coli* strains were grown in Luria Bertani (LB) medium at 37°C. *X*. *oryzae* pv. *oryzae* strains were grown at 28°C in Peptone Sucrose (PS) medium. The plant mimic XOM2 medium (pH 6.5) was prepared by adding 0.18% D (+) Xylose, 670μM L (-) Methionine,10mM Na-Glutamate, 40 μM MnSO4, 14.7 mM KH2PO4, 5mM MgCl2 and 20mM Fe-EDTA. The concentrations of antibiotics used are rifampicin-50μg/ml; spectinomycin (Sp)-50μg/ml; kanamycin (Km)- 15μg/ml for *X*. *oryzae* pv. *oryzae* and 25μg/ml for *E*. *coli*.

**Table 1:** List of bacterial strains and plasmids used in this study.

Bacterial Strains/Plasmids	Relevant characteristic(s)	Reference/ source
***E*. *coli* strains**		
DH5α	λ^–^ f80d*lacZ*DM15 D(*lacZYA*-*argF*)*U169 recA1 endA hsdR17* (r_K_^–^ m_K_^–^) *supE44 thi-1 gyrA relA1*	Invitrogen
***X*. *oryzae* pv. *oryzae* strains**		
BXO1	Wild type; Indian isolate	laboratory collection
BXO43	*rif*-2; derivative of BXO1	laboratory collection
*pglA*^*-*^	*pglA^-^*:: pK18*mob* rif-2; Km^r^; *pglA^-^*; Km^r^	This study
*pmt*^*-*^	*Pmt^-^*:: pK18*mob* rif-2; Km^r^; *pmt^-^*; Km^r^	This study
*pel*^*-*^	*pel^-^*:: pK18*mob* rif-2; Km^r^; *pel^-^*; Km^r^	This study
*pelL*^*-*^	*pelL^-^*:: pK18*mob* rif-2; Km^r^; *pelL^-^*; Km^r^	This study
*pglA* ^*-*^/pHM1	*pglA^-^*/pHM1; *rif-2*; Km^r^, Sp^r^; derivative of *pglA^-^*	This study
*pglA*^*-*^ /pHM1:: *pglA*	*pglA^-^*/pHM1:: *pglA*; *rif-2*; Km^r^, Sp^r^; derivative of *pglA^-^*	This study
*pmt*^*-*^/pHM1	*pmt^-^* /pHM1; *rif-2*; Km^r^, Sp^r^; derivative of *pmt^-^*	This study
*pmt*^*-*^ /pHM1:: *pmt*	*pmt^-^* /pHM1:: *pmt*; *rif-2*; Km^r^, Sp^r^; derivative of *pmt^-^*	This study
**Plasmids**		
pK18*mob*	pUC18 derivative; Mob^+^ Tra^-^ Km^r^; does not replicate in *X. oryzae* pv. *oryzae*	[19]
pTL1	pK18mob+759 bp of internal fragment of *pglA* gene	This study
pTL2	pK18*mob*+793 bp of internal fragment of *pmt* gene	This study
pTL3	pK18*mob*+767 bp of internal fragment of *pel* gene	This study
pTL4	pK18*mob*+653 bp of internal fragment of *pelL* gene	This study
pHM1	Broad-host-range cosmid vector (13.3kb); Sp^r^	[20]
pTC5	pHM1+ 1853 bp gene fragment containing 1671 bp *pglA* gene	This study
pTC6	pHM1+ 1252 bp gene fragment containing 1224 bp *pmt* gene	This study

### Molecular biology and microbiology techniques

Isolation of genomic DNA was performed as described by Leach et al [21]. Plasmid DNA was isolated either by the alkaline lysis method [22] or by using a Macherey Nagel NucleoSpin Plasmid kit (Duren, Germany). Phusion polymerase (Finnzymes; Thermo Fisher Scientific) was used for PCR amplification where high-fidelity DNA synthesis was required while in all other applications Taq polymerase was used. Restriction digestions were done with fast digest enzymes from Thermo Fischer Scientific (Massachusetts, USA). PCR products andrestriction enzyme-digested DNA fragments were purified using Macherey Nagel PCR clean-up and gel extraction kit and a QIAquick nucleotide removal kit (Qiagen, Hilden, Germany) respectively. Ligation reactions using T4 DNA ligase, agarose gel electrophoresis and transformation of *E*.*coli* were all performed as described previously [22]. Plasmids were introduced into *X*. *oryzae* pv. *oryzae* by electroporation [23].

### Sequencing and analysis

The ABI Prism 3700 automated DNA sequencer (Perkin-Elmer, Foster City, CA, U.S.A.) was used for performing DNA sequencing. The obtained sequences were subjected to homology searches using the BLAST algorithm in the National Center for Biotechnology Information database [24].

### RT-PCR analysis of the expression of pectinolytic genes of wild type Xoo (BXO43)

Total RNA was isolated from PS and XOM2 grown cultures of wild type Xoo (BXO43) by using Trizol reagent (Invitrogen, California, USA) as per manufacturer’s instructions. The quality of RNA was assessed by agarose gel electrophoresis and the RNA was quantified using a spectrophotometer (Nanodrop ND-1000, Thermo Fisher Scientific, Massachusetts USA). 1μg of RNA was used for cDNA synthesis using Quanti Tect Reverse Transcription kit (QIAGEN) as per the manufacturer’s instructions. 2 μl of 5 fold diluted cDNA was subjected to qRT- PCR analysis using DyNAmo Flash SYBR green qPCR kit (Thermo Fisher Scientific) following manufacturer’s instructions using primers designed to amplify approximately 100–150 bp fragments of each gene of interest. Primers used are listed in S1 Table. The reaction was performed on a ViiA 7 Real Time-PCR system (Applied Biosystems, California, USA) using the following conditions. Initial denaturation for 7 mins at 95°C, followed by cycling parameters of 95°C for 10 secs, 60°C for 30 secs (40 cycles). Fold change in the expression of genes in XOM2 media over PS media was calculated by 2^-ΔΔCt^ method using 16SrRNA as the endogenous control. Average fold change of three independent experiments was plotted graphically.

### Generation of pectolytic mutants of *X*. *oryzae* pv. *oryzae*

The pectolytic mutants of *X*. *oryzae* pv. *oryzae* were generated by gene disruption which was achieved by homologous recombination and integration of a suicide vector (pK18*mob*) carrying an internal fragment of the gene. The strategy of obtaining a gene disruption mutant using homologous plasmid integration is depicted in S1 Fig. For *pglA*^-^ mutant generation, a 759-bp internal fragment of the *pglA* gene was PCR amplified using genomic DNA of *X*. *oryzae* pv. *oryzae* and the gene-specific PglA FP/ PglA RP oligonucleotide primer pair (S1 Table). This fragment was subjected to digestion with restriction enzymes *Xba*I and *Hind*III and ligated with *Xba*I and *Hind*III digested pK18*mob* cloning vector to obtain recombinant plasmid pTL1. The plasmid was then electroporated into wild type strain BXO43. The *X*. *oryzae* pv. *oryzae* clones that received the plasmid and integrated it into their genome were identified by selection for kanamycin resistance. Plasmid integration at the correct locus and the consequent gene disruption were confirmed by PCR using a combination of gene-specific flanking primers PglA ICF / PglA ICR and vector-specific primers M13F and M13R. The identity of the PCR products was further confirmed by sequencing. Similarly, for generating *pmt*^-^, *pel*^-^, *pelL*^-^ mutants, a 793 bp internal fragment of the *pmt* gene, a 767 bp internal fragment of the *pel* gene and a 653 bp internal fragment of the *pelL* gene were cloned into the *Xba*I and *Hind*III sites of the pK18*mob* vector to generate recombinant plasmids pTL2, pTL3 and pTL4 respectively. The recombinant plasmids were independently moved into the wild type Xoo strain by electroporation. Mutants were selected and confirmed as mentioned above. To prevent polar effect on downstream genes the gene fragments were cloned in the same transcriptional orientation as the lacZ promoter of pK18*mob* vector so that mutation caused by vector integration does not have a polar effect on downstream genes due to activity of the outwardly directed lacZ promoter.

### Generation of complementing strains

For *pglA*^-^ mutant complementation, a 1853 bp DNA fragment containing the complete coding sequence of *pglA* gene was amplified by PCR using primers PglAFLFP and PglAFLRP and genomic DNA of BXO43 as template. The amplified DNA fragment was cloned into *Hind*III-*EcoR*I sites of the broad host range vector pHM1 [21] to create the recombinant plasmid pTC5. The pTC5 plasmid and the empty pHM1 vector were individually electroporated into the *pglA*^-^ mutant strain and the recombinants were selected using spectinomycin resistance as a marker. The presence of the pTC5 plasmid was confirmed by PCR using a combination of *pglA* gene specific primers (PglAFLFP /PglAFLRP) and vector specific primers (M13F/M13R) and also by the sequencing of PCR products. Similarly, for complementation of the *pmt*^-^ mutant, a 1252 bp DNA fragment containing the 1224 bp *pmt* gene was amplified and cloned into the *Hind*III-*EcoR*I sites of pHM1 vector to generate pTC6. This recombinant plasmid and the empty pHM1 vector were separately electroporated into the *pmt*^-^ mutant and recombinants were selected using spectinomycin as a selectable marker. The presence of the pTC6 plasmid in the spectinomycin resistant clones was confirmed as mentioned earlier. Three independent transformants of pTC5 (for *pglA*) and pTC6 (for *pme*) were tested for restoration of enzymatic activity on plate assays.

### Enzymatic assays

Pectin methylesterase activity was tested by growing the *X*. *oryzae* pv. *oryzae* strains on a PSA plate containing 0.1% pectin as the substrate and staining with 0.05% Ruthenium red dye solution in water for 30 mins followed by destaining with water [25].

The polygalacturonase activity was tested on the substrate Na-polygalacturonic acid. The *X*.*oryzae* pv. *oryzae* strains were grown on a PSA plate containing 0.1% PGA and stained with 0.05% ruthenium red solution followed by destaining with water.

### Virulence assay

Wild type Xoo (BXO43), *pglA*^-^, *pmt*^-^, *pel*^-^ *and pelL*^-^ mutants were grown in PS broth with the appropriate antibiotics till saturation. The cells were pelleted by centrifugation at 6000rpm for 5 mins at room temperature, washed with water and resuspended in sterile MQ to a solution of OD600 = 1. Leaf tips of 40–45 days old plants of greenhouse grown Taichung Native-1 (TN-1) were inoculated with surgical scissors dipped in the bacterial suspension. Lesion lengths were measured 10 days post inoculation.

## Results

### *In silico* identification of genes encoding pectin degrading enzymes of Xoo

Bioinformatics analysis indicated that there are 7 genes encoding pectin degrading enzymes in the Xoo genome including two polygalacturonases (*pglA* and *peh1* [pseudo gene]), one pectin methyl esterase (*pmt*), two pectate lyases (*pelL* and *pel*), one rhamnogalacturonase and one rhamnogalacturonan acetyl esterase. These were identified based on similarity searches to pectin degrading genes of Xcc and Xac. A list of the genes encoding pectinolytic enzymes of Xcc, Xac and Xoo is presented in Table 2.

**Table 2:** Pectinolytic Enzymes: Open Reading Frames (ORFs) present in the Xcc, Xac and Xoo genomes.

Pectinolytic enzyme	Xcc ATC 33913	Xac 306	Xoo KACC10331
**Homogalacturonases**			
Polygalacturonases	• XCC2266 *pglA* • XCC3459 *peh1*	• XAC2374 *pglA* • XAC0661 *peh1*	• XOO2699 *pglA* • XOO3959 *peh1*(pseudogene)
Pectinmethyl estearase	• XCC0121* • XCC2265*	• - • -	• - • - • XOO2696 *pmt*
Pectate lyase	• XCC0122* *pel E* • XCC0644 *pel A1* • XCC0645* *pel A2* • XCC2815 *pel B* • - • -	• - • XAC2986 • - • XAC3562 • XAC2373*(d) • -	• - • XOO0821 *pel* • - • - • - • XOO2265 *pelL*
**Rhamnogalacturonases**			
Rhamnogalacturonan acetylesterase	• XCC0154	• XAC0171	• XOO0265
Rhamnogalacturonases	• XCC3377*(t) • XCC3378*(t) • XCC3379*(t)	• XAC3505* • -	• XOO1078 • —

(d) degenerated orfs (t) truncated orfs

* Orf exclusive to each genome

As homogalacturonan (HG) is the abundant pectic polysaccharide of the cell walls, the HG degrading genes including a polygalacturonase (*pglA*), a pectin methyl esterase (*pmt*) and two pectate lyases (*pelL* and *pel*) were chosen for further study. The SIGNALP 4.1 server predicted the presence of an N-terminal signal peptide for the polygalacturoase (PglA) and the pectin methyl esterase (Pmt) but not for the pectate lyases, Pel and PelL.

### Pectin degrading genes are expressed in both PS and XOM2 media

**Fig 1:**
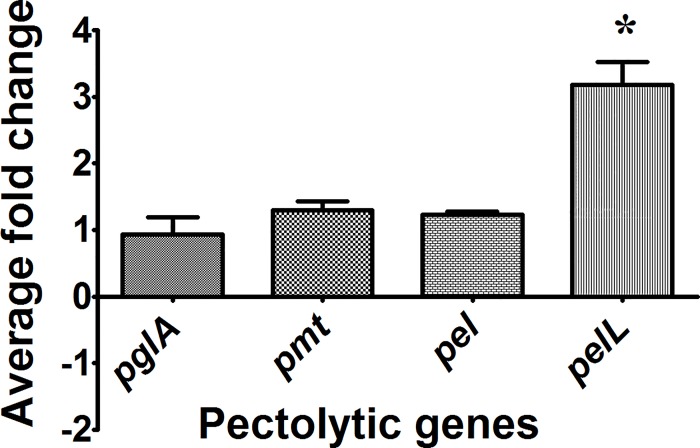
Real Time-PCR analysis of pectin degrading genes. RNA was isolated from the wild type (Wt) Xoo strain grown in either PS or XOM2 medium and converted into cDNA which was further subjected to Real Time-PCR analysis. The expression of the *pglA*, *pel*, *pelL* and *pmt* genes was examined in XOM2 medium using PS as the reference sample and 16SrRNA as an endogenous control. The average fold change (2^-∆∆Ct^) of three independent experiments was plotted for each of the four genes.

In order to check expression of the pectin degrading genes, RNA was isolated from wild type Xoo strain BXO43 which was grown in either nutrient rich peptone sucrose (PS) medium or the plant mimic minimal medium (XOM2). Quantitative Real Time-PCR was performed with the synthesised cDNA using primers designed for each of the four genes chosen for study. In both the media, all the four genes, the polygalcturonase *pglA*, the two pectate lyses *pel* and *pelL*, and the pectin methyl esterase *pmt* were found to be expressed. There was no difference in the expression of *pglA*, *pmt* and *pel* genes by wild type Xoo grown in either PS or XOM2 media. The *pelL* gene was found to be overexpressed (~3 fold) in XOM2 grown Xoo as compared to PS grown Xoo (Fig 1).

Gene disruption mutants were generated for each of these four genes by homologous recombination of pK18*mob* vector having an internal fragment of the gene of interest cloned into it. Mutants were confirmed by PCR and then assessed for pectinolytic activity by growing on medium containing either polygalacturonic acid (PGA) or pectin as substrates.

### Pectin methyl esterase Pmt is the pectin de-esterifying enzyme secreted by Xoo

**Fig 2:**
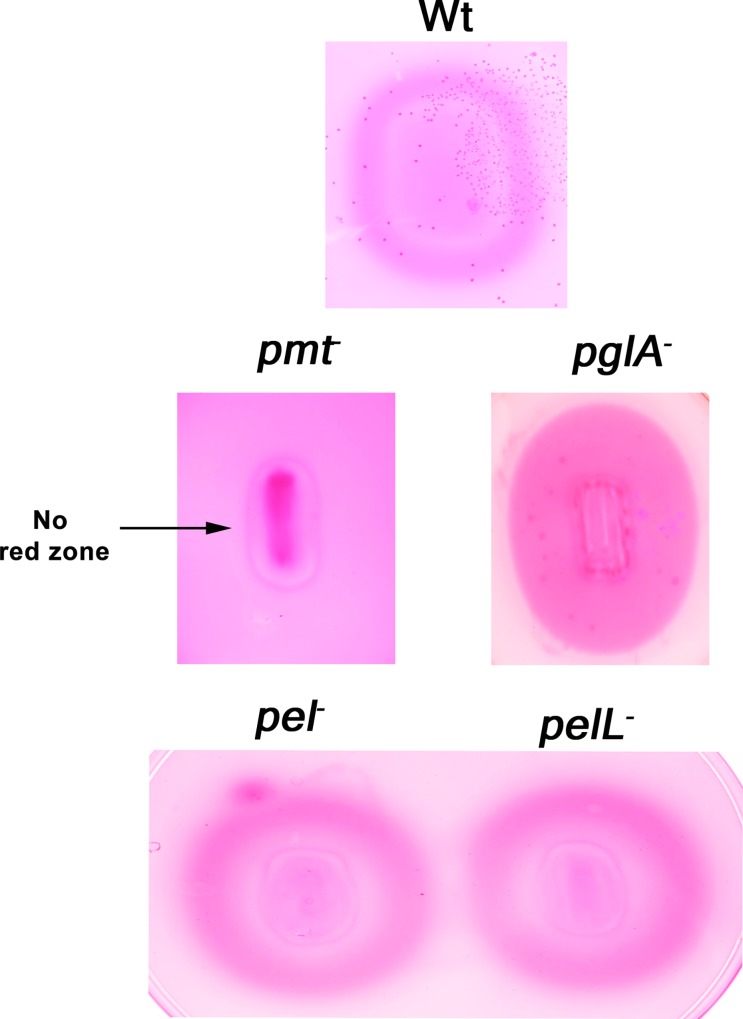
De-esterification of pectin by the secreted pectin methylesterase (Pmt) of Xoo. Wild type (Wt) Xoo and the four mutant strains, namely, *pglA*^-^ (polygalacturonase), *pmt*^-^ (pectin methyl esterase), *pel*^-^ and *pelL*^-^ (both deficient in pectate lyases) were grown on pectin containing medium followed by ruthenium red staining. A red zone around the colony is indicative of de-esterification of pectin. Similar results were obtained in at least three independent experiments

Pectin is the methylesterified form of polygalacturonic acid (PGA). De-esterification of pectin by enzymes favours subsequent action by two types of PGA cleaving enzymes, the polygalacturonases and the pectate lyases. Ruthenium red dye is used for assaying de-esterification of pectin. Pectin de-esterification favours more binding of ruthenium red to PGA leading to formination of a red zone. The red zones were formed around colonies of the wild type, *pglA*^-^, *pel*^-^ and *pelL*^-^ mutants but not around colonies of the *pmt*^-^ mutant. This clearly indicates that de-esterification of pectin is carried out by the pectin methyl esterase Pmt. The wild type, *pel*^-^ and *pelL*^-^ mutant strains further showed a halo within the red zone due to the polygalacturonase activity on de-esterified pectin by their PglA enzyme whereas the *pglA*^-^ mutant showed only the red zone without any clearance within the zone (Fig 2). The complementing strain (*pmt*^-^*/*pHM1+ *pmt)* expressing the Pmt protein from the overexpression vector pHM1 restored the pectin de-esterifying activity and thus formed a red zone like wild type strain. The mutant strain carrying the empty pHM1 vector did not produce a red zone and was in this respect similar to the *pmt*^-^ mutant strain (Fig 4B). Three independently isolated transformants carrying the complementing clone were tested and all three were found to restore the pectin de-esterifying activity.

### PglA is the major pectin degrading enzyme secreted by Xoo

**Fig 3:**
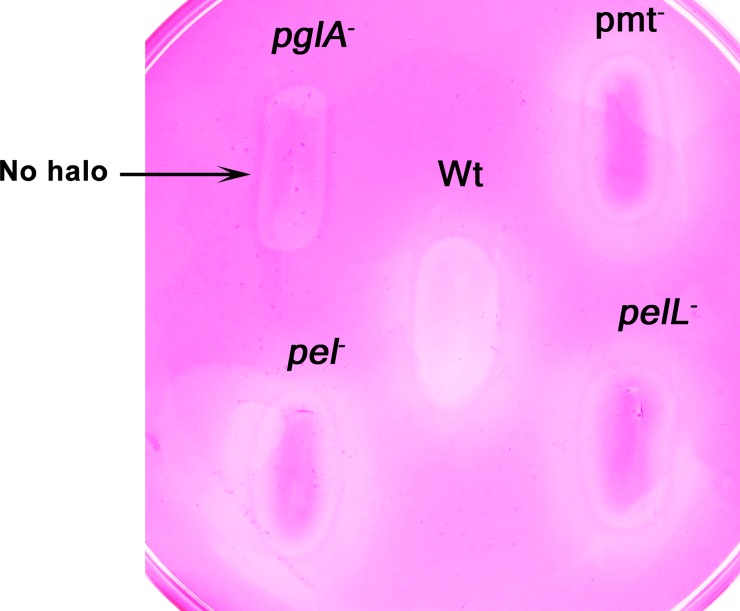
PglA is the major pectin degrading enzyme secreted by Xoo. Wild type (Wt) Xoo and the four mutant strains, namely, *pglA*^**-**^ (polygalacturonase), *pmt—*(pectin methyl esterase), *pel*^**-**^ and *pelL*^**-**^ (both deficient in pectate lyases) were grown on polygalacturonic acid containing PSA medium, followed by ruthenium red staining. A halo around the bacterial colony is indicative of secretion of an active PGA degrading enzyme. Similar results were obtained in at least three independent experiments.

**Fig 4:**
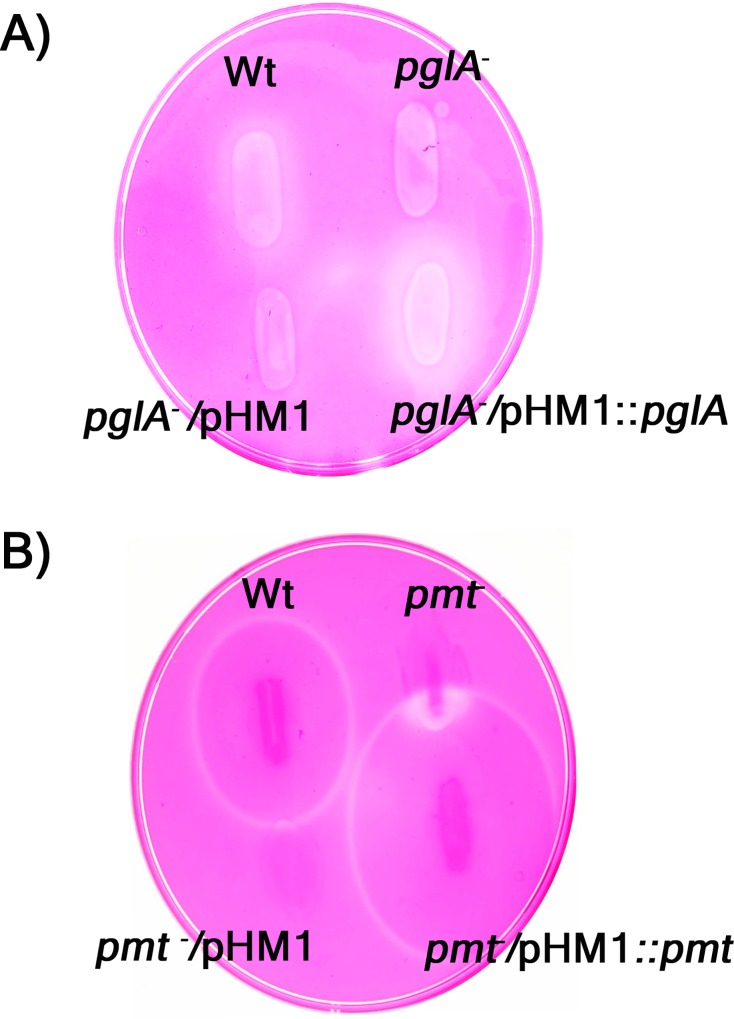
Restoration of enzymatic activities by complementing strains. A) The Wild type (Wt) Xoo strain, the *pglA*^**-**^ mutant, the *pglA*^**-**^ mutant with empty pHM1 vector and the *pglA*^**-**^ mutant with pHM1 having the *pglA* gene were patched on polygalacturonic acid containing PSA medium followed by ruthenium red staining. A halo around the bacterial growth is indicative of active PGA degrading enzyme secreted by the strain. B) The wild type (Wt) Xoo strain, the *pmt*^**-**^ mutant, the *pmt*^**-**^ mutant with empty pHM1 vector and the *pmt*^**-**^ mutant with pHM1 having *pmt* gene were patched on pectin containing PSA medium followed by ruthenium red staining. A red zone around the colony is indicative of de-esterification of pectin. Similar results were obtained in three independent experiments. For each complementing clone, three independent transformants were tested for restoration of enzymatic activity and all of them showed complementation.

The wild type Xoo strain and the *pglA*^-^, *pmt*^-^, *pel* and *pelL*^-^ mutants were tested for their pectolytic abilities on the PGA substrate. The wild type (Wt), *pmt*^-^, *pel*^-^ and *pelL*^-^ mutants acted on PGA and produced a halo. The complete inability of the *pglA*^-^ mutant to act on PGA and produce a halo confirms that polygalacturonase (PglA) is the major pectin degrading enzyme secreted by Xoo (Fig 3). The complementing clone (*pglA*^-^*/*pHM1+ *pglA)* restored the polygalacturonase activity while the empty vector control strain (*pglA*^-^*/*pHM1) did not show any activity on PGA (Fig 4A). Three independently isolated transformants carrying the complementing clone (*pglA*^-^*/*pHM1+ *pglA)* were tested and all three were found to restore the pectin degrading activity.

### Pectolytic enzymes have a minimal role in virulence of Xoo on rice

To assess the role of these pectolytic enzymes in the pathogenicity of Xoo, virulence assays were performed with wild type and the *pglA*, *pmt*, *pel*^-^, *pelL*^-^ mutants by clip inoculation of leaves of the susceptible rice variety TN-1. The length of lesions (indicative of disease progression) formed by wild type and mutants were measured 10 days post inoculation. In some experiments, the mutants displayed a partial virulence deficiency, while in a few other experiments there was no statistically significant difference in lesion lengths formed by the mutant strains as compared to the wild type Xoo strain. The results of virulence assays indicate that the ability to degrade pectin is not a key virulence feature of this pathogen (Table 3).

**Table 3:** Pectolytic enzymes have a minimal role in virulence of Xoo on rice.

Experiments	Wt	*pglA*^-^	*pmt*^-^	*pel*^-^	*pelL*^-^
**1**	**10.54+**/2.91	**11.46+**/2.44	**10.69+**/3.60	**11.20+**/3.38	**11.96+**/2.69
**2**	**6.61+**/-1.52	**3.82+**/1.37*	**3.93+**/0.82*	**3.39+**/-0.72*	**4.1+**/-0.82*
**3**	**6.35+**/-1.54	**4.37+**/0.94*	**4.26+**/1.42*	**4.83+**/-0.90*	**4.78+**/-0.91*
**4**	**8.16+**/-1.71	**7.5+**/-1.46	**7.32+**/-1.85	**7.37+**/-1.48	**7.45+**/-2.08
**5**	**6.23+**/- 1.4	**3.825+**/0.98*	**4.22+**/1.03*	**3.85+**/-1.30*	**3.65+**/-0.95*

* Lesion lengths are significantly different at P < 0.05 level as compared to lesions produced by the wild type strain

## Discussion

The secreted cell wall degrading enzymes like cellulases, xylanases and esterases are important virulence factors of Xoo. Although pectin is a component of the rice cell wall, the role of pectinases in virulence of Xoo has not been assessed. In this study, we have identified the major homogalacturonan degrading enzymes secreted by Xoo and determined the role of these enzymes in virulence. By homology searches, the genes for four homogalacturonases (PglA, Pmt, Pel and PelL) were identified. Gene expression analysis of wild type Xoo has clearly shown that the expression of the *pmt*, *pglA* and *pel* genes remains the same in both PS and XOM2 media. However, the expression of the *pelL* gene was induced in XOM2 medium. The polygalacturonase (*pglA*) gene has a plant inducible promoter (PIP) box in its promoter region and so it is surprising that its expression is not induced in the plant mimic medium [26]. Enzyme activity assays performed with the mutants clearly indicated that PglA is the major pectinase secreted by Xoo and that de-esterification of pectin is carried out by the pectin methyl esterase Pmt.

The virulence assays performed with pectinase deficient mutants of Xoo suggest that they have only a minimal role in promoting virulence on rice. In some experiments, the *pglA*^-^, *pmt*^-^, *pel*^-^ and *pelL*^-^ mutants were partially affected for virulence while in some other experiments the mutants were as efficient as the wild type strain in causing disease. It is not clear why these effects are observed in some experiments and not in others. It is possible that there are some differences in the experimental conditions that we have not been able to control which affect the outcome of the experiment. However, in previous studies on mutants defective in other cell wall degrading enzymes of Xoo (such as a cellulase, a cellobiosidase, a xylanase and a lipase/esterase), we have observed virulence deficiencies in all experiments [17, 18]. Therefore, overall, the homogalacturonases do not appear to be as important for Xoo virulence as the cellulases (ClsA and CbsA), the xylanase (Xyn) and the esterase (LipA) that have been studied earlier.

It has already been reported that pectate lyases and polygalacturonases produced by some of the Xanthomonads are not required for disease development. Dow *et al*. (1989) have shown that inactivation of one of the three isozymes of Pel of Xcc strain 8004 does not affect its ability to cause black rot in turnip plants [12]. The *pehA* mutant of Xcc has been shown to have only a minor role in virulence [2]. Beaulieu *et al* (1991) have demonstrated that both pectolytic and non pectolytic strains of Xcv are equally capable of evoking disease symptoms in host plants [27]. The water-soaked symptom on the margins of canker lesions was related to the expression of the *pel1* gene of Xac [15]. Different pathovars of *Xanthomonas campestris* like *X*. *campestris* pv. *campestris*, *X*. *campestris* pv. *vesicatoria*, *X*. *campestris* pv. *malwacearum*, *X*. *campestris* pv. *glycines* which do not cause soft rot diseases in growing plants are able to macerate potato tuber slices and pepper fruits [28]. This suggests the possibility that the *Xanthomonas* pathogens may, at some time in their life cycle, macerate fruits, tubers or other plant tissues (on their host or on a non host) and that this could be a selection for maintaining and expressing pectinolytic genes within their genomes. We cannot also rule out the possibility that polygalacturonases and pectate lyases are functioning redundantly in causing disease on rice. To address this issue, all four pectin degrading enzymes of Xoo need to be mutated in a single strain and the effect on virulence needs to be assessed. Furthermore, the role of the other class of galacturonases, the rhamnogalacturonases (RGs) on Xoo virulence needs to be addressed.

Pectinases and the products of their actions on plant cell walls, the oligogalacturonides, are well known for their ability to induce defense responses. The pectate lyase (XagP) of *Xanthomonas axonopodis* pv. *glycines* which causes bacterial pustule disease of soybean induces hypersensitive response in tobacco [29]. It was also reported that, the modified pectinolytic enzyme PelI3 of *E*. *chrysanthemi* strain 3937 when infiltrated into tobacco leaves produces a rapid necrotic response [30]. Purified pectate lyase and polygalacturonase of *E*.*carotovora* subsp. *carotovora* induced the expression of defense genes in tobacco and provided resistance to the same pathogen during subsequent infection and it was further shown that this effect is enhanced when the treatment includes a combination of pectic enzymes and a cellulase [31]. Can Xoo secreted polygalacturonases and pectate lyases induce immune responses in rice? We have not tested the ability of purified pectinases to elicit immune responses in rice. However, using an *in vivo* assay with a specific mutant, we have shown that the major polygalacturonase of Xoo (PglA) is not required for inducing defense responses during infection [32]. This is in contrast to the observation that two cellulases (ClsA and CbsA) and a xylanase (Xyn) are required for the elicitation of immune responses during infection. This could be due to the relatively lower content of pectin, as compared to the content of cellulose and xylan in rice/monocot cell walls. Overall, the results presented here on the role of pectin degrading enzymes on virulence and the results of Tayi *et al* [32] on the role of PglA in induction of defense responses during infection suggest that this class of enzymes has a relatively minor role in the *in planta* interaction between Xoo and rice. This is in contrast to the apparently more important role of cellulases and xylanases in promoting Xoo virulence and in induction of rice defense responses.

## Supporting Information

S1 Fig The strategy for generating gene disruption mutants. An internal fragment (green bar) of the gene of interest (orange bar) is cloned into the suicide vector, pK18*mob*. Homologous recombination between the internal fragment of the gene and the chromosomal copy of the gene results in integration of the plasmid into the chromosome and gene disruption.
(TIF)

S1 Table List of primers used in the study. (DOCX)
