# Supplementary figures and images for: Identification of Pectin Degrading Enzymes Secreted by Xanthomonas oryzae pv. oryzae and Determination of Their Role in Virulence on Rice
Source: PLoS One. 2016 Dec 1;11(12):e0166396. doi: 10.1371/journal.pone.0166396 (PMC5132194; doi:10.1371/journal.pone.0166396)

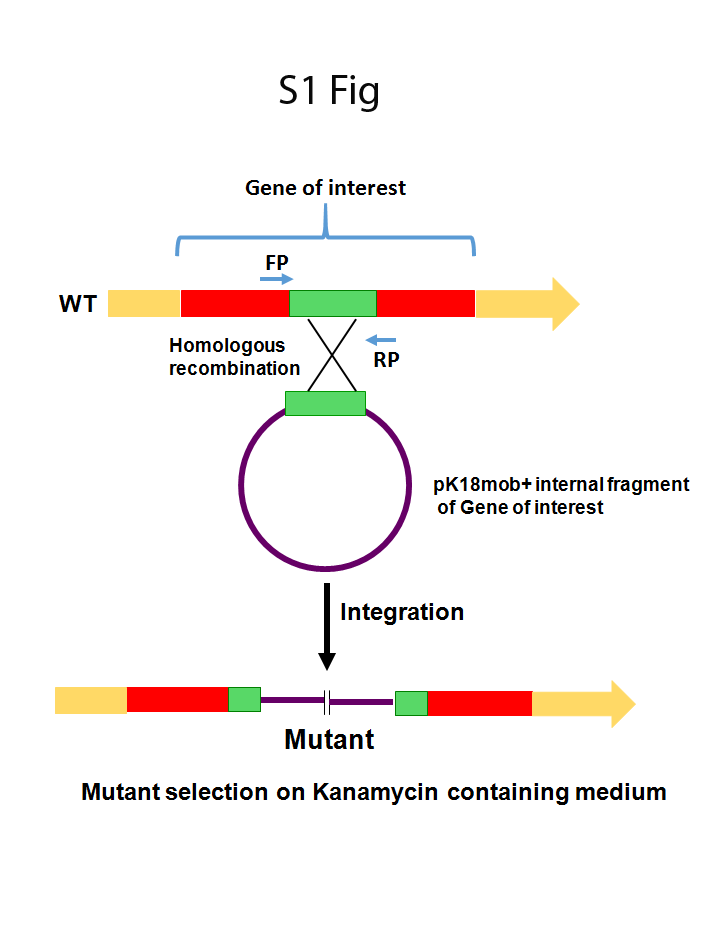

Supplement: S1 Fig — An internal fragment (green bar) of the gene of interest (orange bar) is cloned into the suicide vector, pK18mob. Homologous recombination between the internal fragment of the gene and the chromosomal copy of the gene results in integration of the plasmid into the chromosome and gene disruption. (TIF) [file pone.0166396.s001.tif]
