# Supplementary material for: Identification of Pectin Degrading Enzymes Secreted by Xanthomonas oryzae pv. oryzae and Determination of Their Role in Virulence on Rice
Source: PLoS One. 2016 Dec 1;11(12):e0166396. doi: 10.1371/journal.pone.0166396 (PMC5132194; doi:10.1371/journal.pone.0166396)
Supplement: S1 Table — (DOCX) [file pone.0166396.s002.docx]

**S1 Table. List of primers used in the study.**

| **Primer name** | **Sequence in 5’-3’ direction** |
| --- | --- |
| PglA FP | TGCTCTAGAGCAAAGCGGTGTCACGCTATGGAT |
| PglA RP | CCCAAGCTTGGGTTGAAGCCATCGATGCTC |
| PglA ICFP | ATGTGCCGATAGATCCATGCGT |
| PglA ICRP | AGTGGCTGGATGCACGTCAA |
| Pmt FP | TGCTCTAGAGCAGCTGGTGACCGACAATTGGAATCCC |
| Pmt RP | CCCAAGCTTGGGAACCAGACCTACCTTGGGCGT |
| Pmt ICFP | ATGCAACGTCACCATCCCAACT |
| Pmt ICRP | TGAATTCGCCAAGGCTGGATGA |
| Pel FP | TGCTCTAGAGCATATCAGCGAAGGCAAGCCTTACAC |
| Pel RP | CCCAAGCTTGGGACGACAGCGCCTTGCCATT |
| Pel ICFP | TCACCGTACCAACGGATTTGGA |
| Pel ICRP | TCTGGATCGAGACCATCAGCTACA |
| PelL FP | TGCTCTAGAGCATCCGTCCATACGTTGCGCTAACA |
| PelL FP | CCCAAGCTTGGGCGTTGAAGGGCTCACCGGTAT |
| PelL ICFP | ACCGGCAAGAATTGCCCTGATA |
| PelL ICRP | AATCCCTGCCAACCCTCTAGAACA |
| M13FP | GTAAAACGACGGCCAGT |
| M13RP | GGAAACAGCTATGACCATG |
| PglA FLFP | CCCAAGCTTGGGAAACGTTGGTGTTGCGATCCAG |
| PglA FLRP | CCGGAATTCCGGGGTGTCTCAATCAGCTGTGCTCA |
| Pmt FLFP | CCCAAGCTTGGGACATTGCACAAGCGTATTGACCT |
| Pmt FLRP | CCGGAATTCCGGCACAGCGACGGTCTAATTCCCA |
|  | **Real Time Primers** |
| PglA RT FP | TCGCTGGGTTCCAAGGCATT |
| PglA RT RP | GGCTTTGCGAACGAGACCTTT |
| Pmt RT FP | TGTTCGACAATGCCCTCATCC |
| Pmt RT RP | GCCACAAAGCGGCTATTGATCG |
| Pel RT FP | TGCGTTTCGGTAAGGTGCATCT |
| Pel RT RP | ACCGCTGATCTCGAACACATTACG |
| PelL RT FP | GCGCATCGAAATGATCAACC |
| PelL RT RP | TCGTCCAGGCTGACGAAAT |
| 16S rRNA FP | CCCTAAACGATGCGAACTGGATGT |
| 16S rRNA RP | AGTTTCAGTCTTGCGACCGTACTC |

The underlined sequences indicate engineered restriction sites in primers
